# Cholera outbreak and associated risk factors in Dollo Ado district, Ethiopia: un-matched case-control study, 2023

**DOI:** 10.3389/fepid.2025.1480230

**Published:** 2025-03-31

**Authors:** Fitsum Hagos, Habtamu Molla Ayele, Eyob Hailu Kebede, Abdulnasir Abagero, Awgichew Kifle

**Affiliations:** ^1^ Public Health Emergency Management Directorate, Ethiopian Public Health Institute, Addis Ababa, Ethiopia; ^2^ Maternal and Child Health Directorate, Federal Ministry of Health, Addis Ababa, Ethiopia; ^3^ Public Health Department, Bonga University Health Science College, Bonga, Ethiopia; ^4^ Ethiopian Field Epidemiology Training Program, Ministry of Health, Addis Ababa, Ethiopia; ^5^ Department of Preventive Medicine, School of Public Health, Addis Ababa University, Addis Ababa, Ethiopia

**Keywords:** cholera outbreak, Dollo Ado district, Liben zone, Somali region, Ethiopia

## Abstract

**Background:**

Cholera is a highly contagious bacterial disease that causes severe watery diarrhea. It spreads mainly through contaminated food or water containing *Vibrio cholerae* O139 and remains a major global public health threat. We investigated an outbreak to identify its cause, source, and risk factors and to develop control measures.

**Method:**

A suspected case was classified as the occurrence of acute watery diarrhea in a Dollo Ado District resident aged 2 or older between February 2, 2023 and March 15, 2023. A confirmed case was a suspected case with *Vibrio cholerae* detected in the patient's stool sample. An investigation of the outbreak was conducted; cases were described and the environment, where contamination may take place assessed and an unmatched case-control study conducted in Suftu Kebele, which served as the epi center of the outbreak. Logistic regression was used to identify risk factors for cholera infection.

**Results:**

A total of 92 cases were identified, including 66 males and 26 females, with four deaths (4.3% fatality rate). Males had a higher attack rate (2.4 per 1,000 people) than females (1.6 per 1,000 people). Suftu village was the hardest-hit area (attack rate: 41 per 1,000 people). The outbreak began after a person suspected of having cholera returned from mandera, kenya, on February 2, 2023. Five days later, cases emerged in suftu village. Many residents practiced open defecation and used the dawa river for bathing, washing clothes, and drinking. Using untreated river water significantly increased the risk of infection (AOR = 20, 95% CI: 5.2–73).

**Conclusion:**

The outbreak likely started at a funeral of a suspected cholera case, spreading through contaminated river water. It was contained within a week by restricting river water use and preventing further contamination.

## Introduction

Cholera is a highly infectious bacterial disease that leads to severe, acute watery diarrhea. It is mainly spread through the ingestion of contaminated food or water that contains the bacterium *Vibrio cholerae* O1 or O139, and is considered a significant threat to global public health. On average, 3–5 million cases and over 100,000 deaths from cholera occur worldwide each year (1). This illness tends to spread more readily in nations where living conditions are substandard, including a lack of access to safe water and basic sanitation facilities (2).

The global burden of cholera is estimated at 1.3–4.0 million cases and 21,000–143,000 deaths annually. Approximately 30% of these cases and 80% of the fatalities occur in Africa, where the continent experienced the highest number of cholera outbreaks in recorded history in 2021, with 19 countries reporting more than 137,000 cases and 4,062 deaths (3, 4).

Each year, nearly 70 million people in Ethiopia are at risk of contracting cholera, with an estimated 275,221 cases and 10,458 deaths occurring annually, resulting in an incidence rate of 4 cases per 1,000 populations (5).

By understanding the risk factors associated with cholera outbreaks, it becomes possible to identify practices that put communities at risk of infection, which in turn enables the design of effective behavior change interventions (6). Studies conducted in different parts of the world have identified known risk factors for cholera outbreaks, such as contaminated water sources, unwashed raw vegetables, inadequate latrines, and heavy rains and floods (7). Although no nationwide representative study has been conducted in Ethiopia to identify risk factors associated with cholera outbreaks, limited studies have identified various risk factors, including contact with cholera cases from unsanitary latrines and travel history to cholera outbreak areas (8).

To date, cholera outbreaks have been reported in 14 districts across the Oromia and Somali regions of Ethiopia. Since January 1, 2023, Ethiopia has reported a total of 1,597 cholera cases with 38 deaths (CFR = 2.4%). As of February 22, 2023, the Somali region, which was previously reported to be under control, has active transmission, with a coverage of 14,021 (43.9%) (9).

The dollo ado district public health emergency management reported an acute watery diarrhea case on February 2, 2023, which was later confirmed to be cholera. The aim of this study is to investigate the outbreak, identify its etiology, source, and risk factors, and develop strategies to control the outbreak.

## Methods and materials

### Study area and period

**Figure 1:**
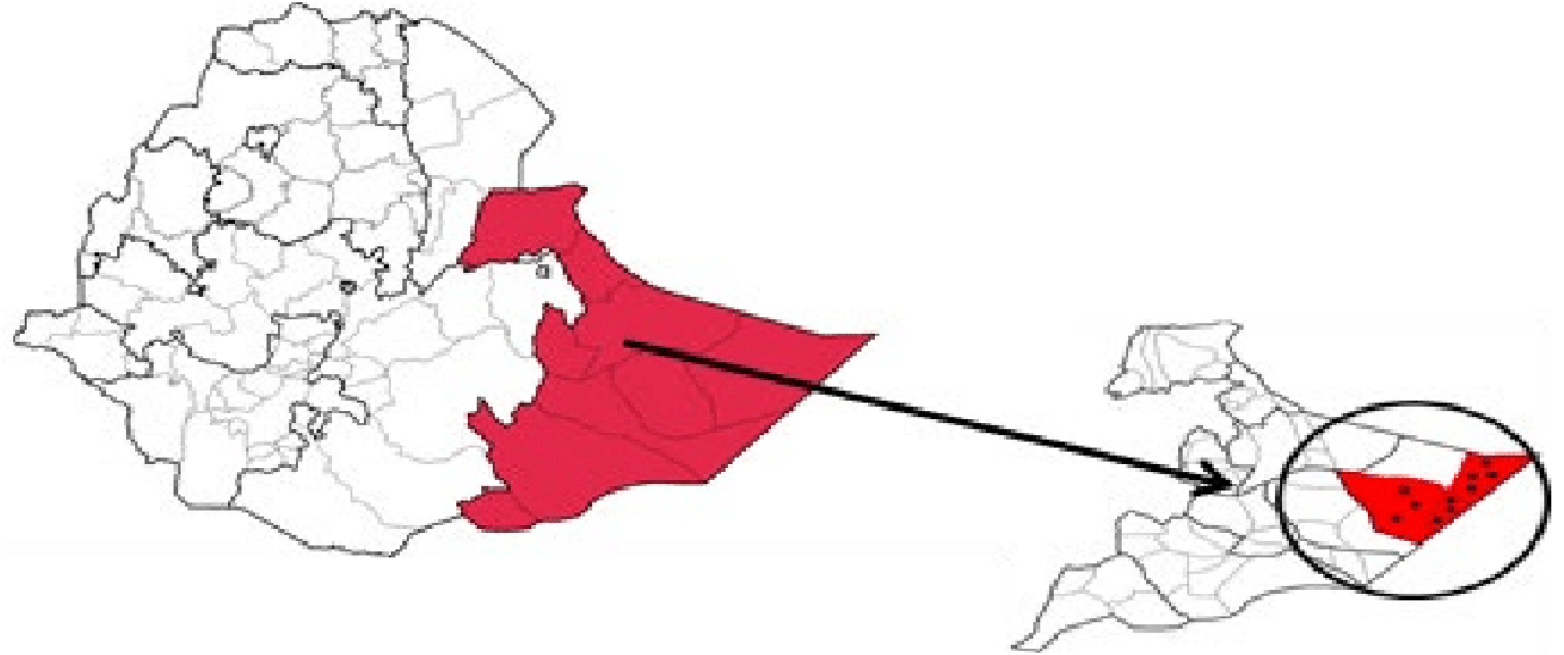
Location of Dollo Ado district in Ethiopia (source: https://pubmed.ncbi.nlm.nih.gov/28983395/).

The study was conducted in dollo ado district, Somalia Region, from February 02 to March 15, 2023 (Figure 1).

### Case definition and finding

A suspected cholera case was defined as the onset of acute watery diarrhoea between February 2nd and march 15th, 2023, in a resident of the suftu district who was at least 2 years old. A confirmed cholera case was defined as a suspected case in which Vibrio cholerae was isolated from the stool sample through culture testing. This classification follows standard cholera case definitions, where all confirmed cases originate from suspected cases based on clinical presentation before laboratory confirmation (10). In this study, unmatched case-control study design was conducted. Cases included individuals who presented with cholera and acute watery diarrhoea with moderate or severe dehydration in the dollo ado district during the study period. Cases were included if they were at least 2 years old, had a positive result in a rapid diagnostic test for cholera, and agreed to participate in the study. Cases were excluded if they lived outside of the Dollo Ado district after the start of the cholera outbreak. Controls were selected from the same neighbourhood as the cases among houses that had not reported any cholera cases since the beginning of the outbreak. Individuals who were at least 5 years old, had lived in the same neighbourhood as the cases since the beginning of the outbreak up to March 15th, 2023, did not have three liquid watery stools within 24 h at any time since the beginning of the outbreak, and agreed to participate in the study were included as controls. Controls were specifically chosen from individuals residing in the house directly to the left of the case's residence. If a suitable control was not found in that house, the data collector moved to the next house on the left. Controls were excluded if they had ever lived outside of the district after the outbreak began. Cholera risk factors were defined as any event or behaviour related to water and food consumption, as well as hygiene practices of people living in Dollo Ado that could potentially increase the likelihood of contracting cholera.

### Data collection

Data was collected between February 2, 2023, and March 15, 2023, by well-trained health workers using a semi-structured questionnaire. The questionnaire was translated into Somali by the interviewers who participated in our pilot test, and the final version was used for face-to-face interviews with cases and controls.

Throughout the study period, cholera cases were not evenly distributed, with notable fluctuations in case numbers. Peaks were observed at The peak of the outbreak occurred on February 5 reflecting potential clusters of transmission or changes in exposure patterns. These variations were considered in our analysis to better understand the outbreak dynamics.

Interviewers collected data from cases or their caretakers at cholera treatment centres (CTCs) after reviewing the registers for admitted cases and also visited the homes of cases to gather information on water, sanitation, hygiene, and food consumption. Two controls were selected for each case from neighboring houses. The questionnaires collected demographic details such as age, gender, address, neighborhood, street, and occupation, as well as clinical information, including the date of diarrhea onset, symptoms, and diagnosis. Additionally, data on travel history, contact with infected persons, hygiene practices, eating outside the home, and attendance at gatherings were gathered. Information on household water sources—including public wells, truck-delivered water, private wells/boreholes, and stored water containers—was also recorded.

### Descriptive epidemiology

The attack rate was calculated by place (district, sub-county, and village), age (categorized as 2-4, 5-14, 15-29, 30-59, and ≥60 years), and sex using the 2023 population estimates of persons aged two and above obtained from Ethiopian Statistical Services (ESS). The age groups were chosen based on epidemiological patterns observed in previous cholera outbreaks, where younger children (2–4 years) are at higher risk due to poor immunity, school-aged children (5–14 years) have increased exposure through schools and communal activities, and adults (15–29, 30–59, and ≥60 years) may have varying risks due to occupational and environmental factors. This categorization allows for a more precise understanding of age-related risk factors and transmission dynamics.

### Environmental survey

An environmental survey of households of cases and controls was undertaken. Their sources of water supply were inspected, principally observing activities around the community-wide Dawa River, the drainage system, and general sanitation along the water bodies, and collected water specimens and sent them for water quality testing at the Regional Hospital laboratory.

### Sample collection and processing

Stool samples were collected from the initial four cholera case-cases and transported to the laboratory using Cary-Blair transport media to ensure sample stability. Additionally, 500 ml water samples were collected from Dawa river water in sterile containers and analysed at the Regional Hospital laboratory aided by the World Health Organization.

To process water samples, 0.22 μm membrane filters were used to filter the collected water. The filters were then enriched in alkaline peptone water (pH 8.4) and incubated at 37°C for 4–6 h before culturing on selective media, following previously established methods. Stool samples underwent the same enrichment in alkaline peptone water and culturing procedures.

### Isolation and identification of V. cholerae

Following incubation on selective media, colonies with characteristic V. cholerae morphology were isolated and further identified as per established protocols. To determine serogroups, polyvalent antisera slide agglutination test was used, and specific serological subtypes (Inaba and Ogawa) were identified through monovalent antisera for subtype verification.

### Hypothesis generation

Ninety-two individuals, identified through active case searches, were interviewed. These individuals’ data was collected by the interviewers from either the cases themselves or their caretakers at the CTCs, after reviewing the registers of admitted cases. To gather additional information, the interviewers also visited the homes of the case cases to inquire about the food consumed, water and sanitation practices, and overall hygiene. The interview questions covered a range of topics, including the source of drinking water, the types of food consumed, any recent travel history, and hand washing practices.

### Case-control study

**Figure 2:**
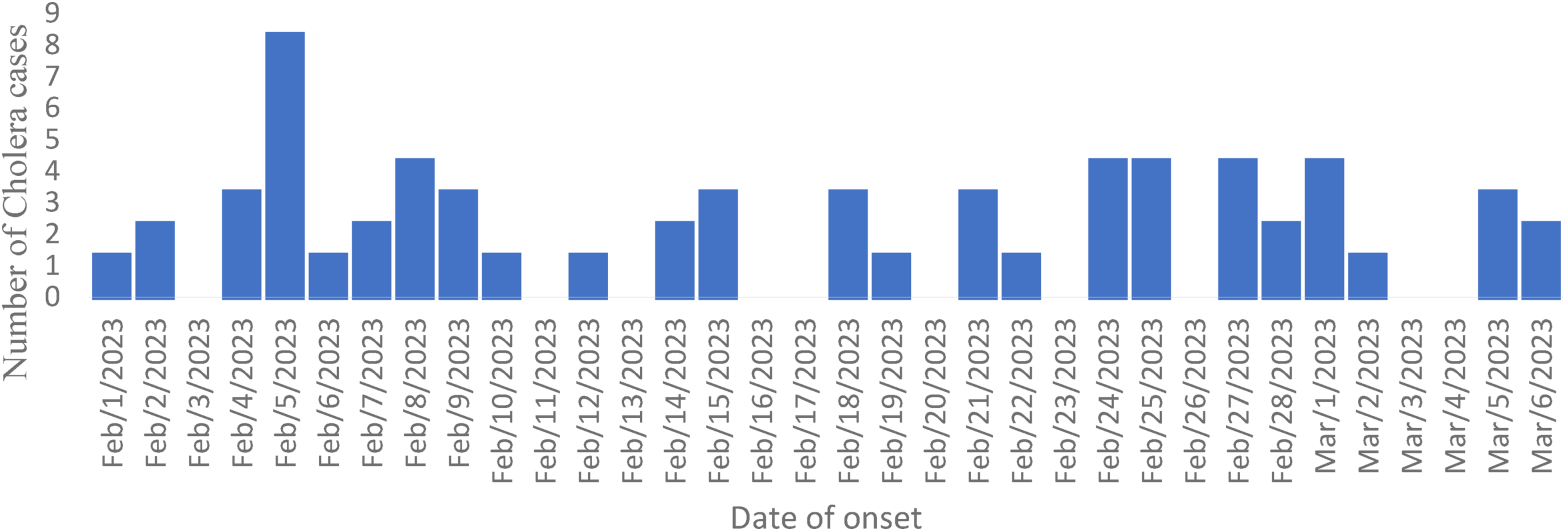
Trend of a cholera outbreak in Dollo Ado district, Somali region, Ethiopia from 01 February to March 11, 2023.

A case-control study was conducted on the most affected village, Suftu 01, in Dollo Ado district, which included 92 cases and 184 controls (Figures 2). All case cases in Suftu village were included in the study. A control was defined as a resident of Suftu village aged ≥2 years with no history of acute watery diarrhea from February 02–March 15, 2023. Households without any cases were considered as controls and systematically sampled. Two control individuals were randomly selected from each sampled household using the lottery method. A questionnaire was administered to both case and control individuals to gather their demographic data (age, sex), clinical characteristics (onset date, symptoms, healthcare seeking behavior), and exposures (water sources, travel history, funeral attendance, food and water preparation). Logistic regression was used to analyze the data and generate odds ratios and 95% confidence intervals for factors associated with cholera infection. In addition, a common reference group analysis was conducted for factors that were significant at a bivariate level to assess the differences in the odds of infection based on combinations of the factors associated with cholera infection.

## Results

### Descriptive epidemiology

A total of 92 cholera cases were reported in Dollo Ado District, Somali Region (AR = 20/100,000), with four deaths, resulting in a case fatality rate (CFR) of 4.3%. The median age of the affected individuals was 24 years (IQR: 15–37). Males had a higher attack rate (AR = 11/10,000) than females (AR = 5/10,000). The most affected age group was 30–59 years (Table 1).

**Table 1:** Attack rates of case cases by sex and age during a cholera outbreak in Dollo Ado district, Somali region, Ethiopia, 2023.

Characteristics	Cases (*n* = 92)	Population	AR/10,000
Sex			
Male	66	60,778	11
Female	26	50,733	5
Age (years)			
2–4	4	12,150	3
5–14	27	41,261	15
15–29	15	30,880	5
30–59	36	20,700	17
>60	10	6,520	15

Five of the seven kebeles in the district reported cases, with Suftu 01 kebele experiencing the highest attack rate (AR = 36/1,000) (Table 2).

**Table 2:** Attack rate by Kebele during a cholera outbreak in Dollo Ado district, Somali region, Ethiopia, 2023.

Name of Kebeles	Total populations	Number of cases	AR/10,000	CFR/100
Suftu 01	13,680	50	36	8
Suftu 02	13,225	29	22	0
Boryale	4,462	7	16	0
Garbo addo	465	2	4.3	0
Dollo ado 02 Kebelle	23,500	4	1.7	0
Total	55,332	92		

The outbreak's index case was a 30-year-old woman from Suftu 01 kebele, reported on February 2, 2023. She had a history of travel to Mandera, Kenya—an area with ongoing cholera transmission—five days before symptom onset. She presented with acute watery diarrhea, vomiting, and severe dehydration and was admitted to Suftu Cholera Treatment Center, where Vibrio cholerae was isolated from her stool sample. She was discharged after recovery on February 10, 2023.The outbreak escalated between February 5 and March 1, 2023, peaking on February 5. Response efforts began immediately, including the distribution of water treatment chemicals.

### Hypothesis generation findings

Interviews with 92 cholera cases, identified through active case search, were conducted at Cholera Treatment Centers (CTCs) and during home visits. The focus was on water, sanitation, hygiene, and food consumption. Many cases had consumed food from local vendors, particularly street foods such as fruits, salads, and raw vegetables. Their primary drinking water sources were untreated or inadequately treated, including wells and rivers, and some households used uncovered containers. Several individuals had recently traveled to areas with poor sanitation and ongoing cholera outbreaks.

Households often lacked proper sanitation, relying on shared or public latrines, and had inconsistent handwashing practices, especially before meals and after using the toilet. The frequent consumption of food from informal vendors with poor hygiene and inadequate food storage conditions was common.

The findings suggest that cholera transmission is linked to poor water quality, inadequate sanitation, hygiene practices, and the consumption of contaminated food. These insights guide further investigations and public health interventions.

### Environmental findings

An environmental survey was conducted in households of both cases and controls, with a focus on water supply sources, community activities around the Dawa River, drainage systems, and overall sanitation along the water bodies. Water samples were systematically collected from multiple locations, including household storage containers, communal water sources, and the Dawa River. A total of 3 water samples were collected, with 1 from household containers, 1 from communal sources, and 1 from the river. The samples were carefully handled following standard procedures, stored in sterile containers at 4°C, and transported under controlled conditions to the Regional Hospital laboratory for microbiological and physicochemical analysis.

### Laboratory findings

Vibrio cholerae O139 Ogawa was isolated from the water samples using standard microbiological techniques. The water samples were first enriched in alkaline peptone water to enhance bacterial growth. Subsequently, they were streaked onto thiosulfate-citrate bile salts-sucrose (TCBS) agar for selective isolation. Suspected colonies were further identified through biochemical tests, including oxidase and string tests, and confirmed using molecular assays such as polymerase chain reaction (PCR) (Table 3).

**Table 3:** Comparing cholera exposure characteristics among cholera cases and controls using bivariate analysis, Dollo Ado district, Somali region, Ethiopia.

Exposure	Cases no %	Controls no %	COR	95% CI
Knowing the transmission means of cholera				
Yes	29 (31)	96 (52)	2.38	(1.4–4)
No	63 (69)	88 (48)		
Traveling history 5 days before the onset of illness to the affected area				
No	5 (5)	126 (68)	37.9	(14.6–98)
Yes	87 (95)	58 (32)		
Drinking river water				
Yes	87 (94)	18 (10)	161	(58–449)
No	5 (6)	166 (90)		
Using soaps for hand washing				
Yes	86 (92)	22 (11)	0.01	(0.004–0.02)
No	6 (7)	162 (89)		
Contact history with cholera suspected person				
Yes	35 (39)	117 (64)	0.3	(0.21–0.59)
No	57 (61)	67 (36)		
Usage of latrine				
Yes	3 (3)	88 (49)	28	(8.6–93)
No	89(97)	96(51)		

### Case-control study findings

A case-control study was conducted that included with 92 cases and 184 community-based controls, with median ages of 24 and 26 years, respectively, and standard deviations of 11 years for both groups. Bivariate analysis showed no significant associations between cholera and sex, age, marital status, education, or occupation (Table 2).

In terms of exposure, several factors were statistically associated with an increased risk of cholera. These included recent travel to cholera-affected areas, consumption of river water, and the use of a latrine. Conversely, washing hands with soap and avoiding contact with individuals suspected of having cholera were identified as protective factors. Notably, there was no statistically significant association between water treatment with chemicals and cholera illness.

### Multivariate analysis of risk factors

The multivariable model included variables such as knowledge of cholera transmission, travel history in the 5 days before illness onset, hand washing with soap, latrine use, drinking river water, and contact with a suspected case.

The analysis identified knowledge of cholera transmission (AOR: 4.2, 95% CI: 1.3, 13) and drinking river water (AOR: 20, 95% CI: 5.2, 73.2) as independent risk factors, while hand washing with soap (AOR: 0.13, 95% CI: 0.01, 0.99) was a protective factor (Table 4).

**Table 4:** Comparing cholera exposure characteristics among cholera cases and controls using multivariate analysis, Dollo Ado district, Somali region, Ethiopia.

Exposure	Cases number %	Controls number %	AOR	95% CI
Knowing the transmission means of cholera				
Yes	29 (31)	95 (52)	4.2	(1.3–13)
No	63 (69)	89 (48)		
Using soaps for hand washing				
Yes	86 (92)	20 (11)	0.13	(0.01–0.99)
No 1	6 (7)	164 (89)		
Drinking river water				
Yes 1	87 (94)	18 (10)	20	(5.2–73.2)
No	5 (6)	166(90)		

## Discussion

Cholera is a major public health issue and a telling sign of poor socioeconomic development worldwide. Although it was once prevalent in many regions, it is now primarily confined to underdeveloped nations in the tropical and subtropical zones (11, 12). This finding aligns with a study carried out in a rural area in north-central Nigeria in 2014 (13).

The most affected age group in our study was 15–44 years, with males accounting for 71.7% of cases, which is consistent with a study from Ghana (14).

The case fatality rate (CFR) in our study was 4%, lower than previous studies in Oromia (4.11%) and the Afar region (4.4%) (15). This difference may be attributed to better healthcare access, enabling more timely treatment

The attack rate in the district was 1.6/1000, which is higher than the study conducted in the Afar region (8.5/1,000) but with a lower fatality rate of 4.4%. This discrepancy may be due to the fact that cases in the Afar region had to travel further to reach health facilities, leading to delayed treatment (8).

Our study identified drinking water from public rivers as a significant risk factor for cholera, a finding consistent with studies in Iran and Nigeria (16). Contaminated water sources have long been recognized as major contributors to cholera outbreaks (17). In their review of global cholera outbreaks from 1995 to 2005, researchers identified water source contamination, heavy rainfall and flooding, and population displacement as the main risk factors for cholera outbreaks. Moreover, an Ethiopian study (5) also showed the association of cholera outbreak with unsafe drinking water. Studies showed that *V.cholerae*, the causative agent of cholera, has been well established as the native flora of aquatic environments rendering drinking water a risk factor for cholera outbreaks (18). Cholera outbreaks are often caused by the spread of the disease from aquatic environments, such as water systems, which can become contaminated during heavy rainfall and flooding (19). In our present outbreak investigation, we examined river water sources that were utilized by cholera cases and discovered that the water sources were flooded following heavy rainfall. The water supply, distribution, and chlorination system in the Dollo Ado district prevents the spread of cholera through chlorination and daily monitoring during cholera outbreaks. In recent years, most reported outbreaks have not been due to problems with the water supply. Our study found a significant association between knowledge of cholera and cholera infection (AOR, 4.2; 95% CI, 1.3, 13). Compared to controls, cholera cases have been found to travel more in the prior two weeks (*p* < 0.05) (20).

Numerous demographic and socioeconomic factors, including age, gender, social status, economic status, and travel history, have been found to play a crucial role in susceptibility to cholera-causing Vibrio cholera. Inadequate sanitation and limited access to health facilities during travel, as well as purchasing food from street vendors or restaurants, are key factors contributing to cholera infection. It is clear that good sanitation and hygienic practices while traveling can largely prevent infection with the disease (11, 21).

Hand washing with soap and water is essential in protecting against cholera and becomes increasingly important during outbreaks when cholera contamination is widespread, and disease dissemination occurs through multiple vehicles (16).

Our analysis of this cholera outbreak found that washing hands with soap after using the latrine was an independent protective factor against cholera illness (AOR 0.13; 95%CI (0.01,0.99). This finding agrees with previous studies (5), which demonstrated that washing hands with soap before food reduced the risk of diarrhea by nearly 20% during an acute watery diarrhea outbreak investigation in Ethiopia. Additionally, in Ethiopia (8), showed that washing hands with soap before food reduced the risk of diarrhea by 85% during an acute watery diarrhea outbreak investigation. Therefore, consistent and sufficient soap distribution and hand hygiene awareness campaigns would be significant interventions in cholera prevention and control strategies, particularly during outbreak settings.

Furthermore, knowledge and awareness of the general public are essential aspects of cholera outbreak management and prevention (22). Insufficient knowledge and inadequate practices towards cholera can exacerbate its transmission (19). Cholera outbreaks are commonly associated with insufficient water, sanitation, and hygiene (WASH) (23, 24). These conditions are prevalent in regions experiencing political and economic instability, including war and a resurgence of infectious diseases (25). Consequently, the increased burden of infectious diseases can further weaken the fragile healthcare system and public health control measures in the Somali Region, which is already grappling with political turmoil and economic challenges (26).

## Conclusion

The primary causes of cholera transmission in Dollo ado district, Liben Zone were mainly linked to insufficient water and sanitation, hygiene, and a lack of understanding of preventive measures. To address this issue, a health education campaign is strongly recommended to raise public awareness about the importance of daily water chlorination and washing hands before consuming food.

Furthermore, our multivariate analysis revealed that hand hygiene provides protection against cholera, which implies that waterborne exposure may contribute to the transmission of the disease.

## Recommendations

We suggest limiting immigration exposure to nearby areas where cholera has been confirmed. Additionally, vaccinating the mass population may be an option. Boiling water before drinking is also recommended. It is also important to educate the community on proper food hygiene.

During community education campaigns, particularly in outbreak situations, emphasizing the importance of washing hands with soap before eating and preparing food is crucial. To prevent flooding and subsequent water contamination, building flood embankments around drinking water sources is recommended.

## Data Availability

The raw data supporting the conclusions of this article will be made available by the authors, without undue reservation.
